# Novel Endoscopic‐Assisted Long Plate Approach for Mandibular Condylar Fracture Reconstruction—A Preliminary Study

**DOI:** 10.1002/kjm2.70033

**Published:** 2025-05-19

**Authors:** Chao‐Hsin Huang, Chia‐Chen Lee, Yi‐Chia Wu, Hsiao‐Chen Lee, Yur‐Ren Kuo, Su‐Shin Lee

**Affiliations:** ^1^ Division of Plastic Surgery, Department of Surgery Kaohsiung Medical University Hospital, Kaohsiung Medical University Kaohsiung Taiwan; ^2^ Department of Surgery, Faculty of Medicine, College of Medicine Kaohsiung Medical University Kaohsiung Taiwan; ^3^ Regenerative Medicine and Cell Therapy Research Center Kaohsiung Medical University Kaohsiung Taiwan; ^4^ Academic Clinical Programme for Musculoskeletal Sciences Duke‐NUS Graduate Medical School Singapore; ^5^ Faculty of Medicine, College of Medicine, Kaohsiung Medical University Kaohsiung Medical University Hospital Kaohsiung Taiwan; ^6^ Department of Biological Sciences National Sun Yat‐Sen University Kaohsiung Taiwan

**Keywords:** endoscopic, subcondylar, surgery

## Abstract

Mandibular condylar fractures can lead to facial asymmetry, malocclusion, and temporomandibular joint instability. To minimize the risk of these issues, endoscopic‐assisted reduction techniques were developed. Nevertheless, the confined working space inherent in endoscopic procedures poses challenges, especially in cases with unstable fracture sites, movable plates, and screws. To solve this dilemma, we developed a novel surgical technique using long plate for condylar fracture fixation. “Long Plate Technique” involves a long plate being stabilized via the submandibular incision site, with screws inserted through the trocar to affix the titanium plate onto the superior condylar segment from the tragus incision site. A total of 98 patients were included in this study. The overall average operation time was 365 min. Specifically, the average operation time for the group treated with closed reduction and intermaxillary fixation was 250 min, for Group B cases treated with short plate open reduction and intermaxillary fixation was 429 min, and for Group C treated with long plate open reduction and intermaxillary fixation was 413 min. The utilization of the Long Plate Technique provides a secure and efficient operation technique that can lessen the stress on surgeons during the operation.

## 1 Introduction

Mandibular condylar fracture accounts for 20%–54% of the mandibular fractures [1]. Mandibular condylar fracture can cause ramal height difference and temporomandibular joint instability, which in turn impairs functions of the temporomandibular joint such as malocclusion, jaw displacement, movement restriction, and ankylosis. Facial asymmetry can compromise self‐confidence and social interaction. MacLennan et al. classified mandibular condylar fracture based on the relationship between the condyle and the mandible proper [2]. Lindahl et al. et al. also proposed condylar fracture categorization based on the level of fracture, dislocation extent, and condylar head in relationship to the auricular fossa [3]. These classifications act as guidelines for surgical planning, though the surgical management of mandibular condyle fracture remains controversial.

As surgery advancements in the management of the mandibular condylar fracture were made, ongoing debates between closed and open reduction have not come to a conclusion [4, 5, 6]. Closed reduction can result in misalignment healing with malocclusion. Closed reduction maintained the gold standard for decades [7, 8]. Open reduction, on the other hand, has the risk of facial nerve injury and hypertrophic scarring [9]. Open reduction was favored in selected cases such as adults with displaced or dislocated complex fracture [5, 10]. In recent years, numerous comparative studies were conducted, and some showed no significant difference in morbidity rates [6, 11]. In addition, endoscopic‐assisted reduction was developed to minimize complications such as facial nerve injury and simultaneously reach excellent reduction of the subcondylar fracture [12, 13]. Endoscopic‐assisted reduction falls into intraoral approach or extraoral approach. The intraoral approach is constrained by limited visibility and accessibility of the fracture site. The extraoral approach, including periauricular access, retromandibular access, or submandibular access, can cause salivary fistulas, facial nerve traction injury, auricular anesthesia injury, or parotid gland trauma [12]. In addition, fracture site instability, movable screws and plates, and limited working space aggravate the fixation difficulties during operation.

Thus, we presented our cases with a novel endoscopic‐assisted open reduction method called “Long Plate Technique.” This technique solves the dilemma of fixation process and minimizes risk of nerve injury and parotid gland trauma through a combination of periauricular and submandibular access.

## 2 Patients and Methods

**FIGURE 1:**
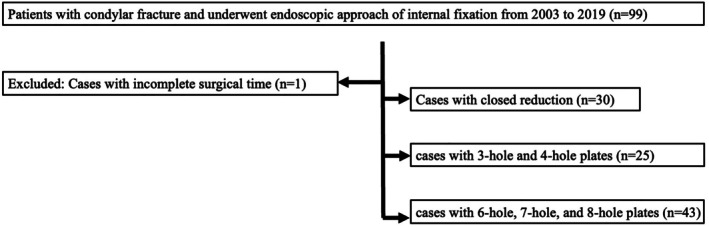
Flow chart of patient inclusion criteria.

The retrospective study was conducted from 2003 to 2019 in a single medical center. The approval from the institutional review board was obtained. The study collected cases from a group of three to five surgeons in the plastic surgery department. Our study included 99 patients older than 18 years with either unilateral or bilateral subcondylar fractures who underwent endoscopic internal fixation. One patient was excluded due to incomplete surgical records or radiographic images (Figure 1), resulting in 98 cases being enrolled in the study. The cases with complete records were categorized into three groups. Group A comprised cases treated with closed reduction and intermaxillary fixation (*n* = 31). Group B consisted of cases treated with open reduction and intermaxillary fixation, utilizing three‐hole and four‐hole plates (*n* = 25). The open reduction was completed via a single preauricular approach. Group C encompassed cases treated with endoscopic subcondylar fixation, employing six‐hole, seven‐hole, and eight‐hole plates (*n* = 42). Data collection encompassed various elements such as demographics, preoperative examinations, operative records, postoperative pictures and data, as well as outpatient clinic charts for follow‐ups. A preoperative survey including computed tomographic scans with selected three‐dimensional image reconstruction was conducted to facilitate surgical planning.

**FIGURE 2:**
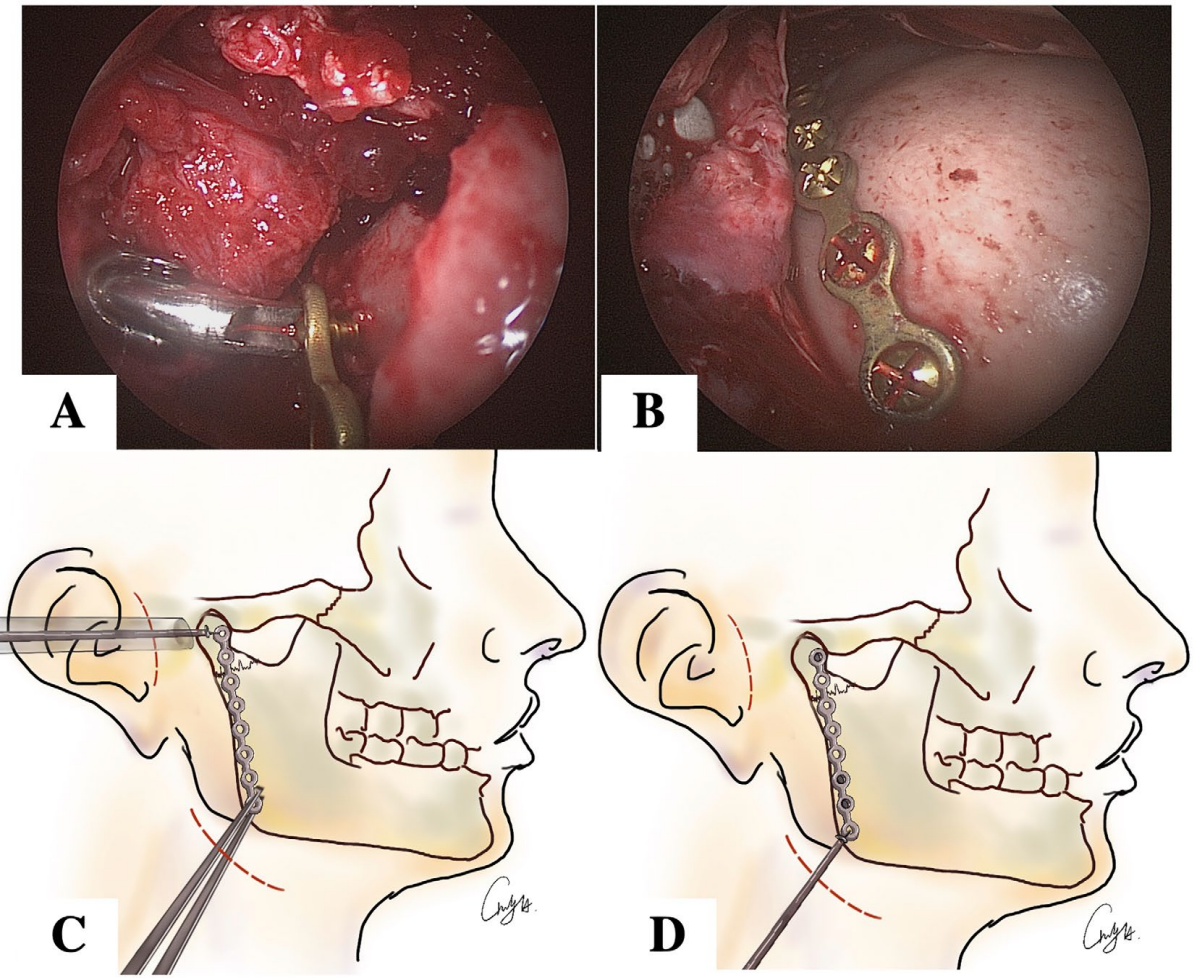
An Illustration of how Long Plate Technique secure insertion of screws. (A) Intraoperative image depicting the drilling of the first hole. (B) Intraoperative image displaying the final appearance of the long plate after fixation; (C) Drilling can be safely done through preauricular incision while a long Mosquito manipulates a long plate; (D) The rest of drilling can be done through submandibular incision.

“Long Plate Technique” started with arch bar fixation of mandible and maxilla (Figure 2). Fracture site exposure through a submandibular incision 1 cm below inferior border of the mandibular ramus. After we cut through platysma and approached to the mandible angle, subperiosteal dissection of the ramus bone was performed. A 30° angled endoscope was inserted first after subperiosteal dissection. The facial flap was flipped upward to expose the fractured condyle neck. Another 1.5 cm incision along the tragus line was made and dissected the tunnel along with the external ear canal to the condylar area. A trocar insertion through tragus incision was monitored. After proper reduction of the displaced condylar segment, a six‐hole, seven‐hole, or eight‐hole titanium plate was selected for internal fixation. The choice of plate was made based on the length of the patient's mandibular ramus. The titanium plate was inserted into submandibular incision until it reached the superior border of the fracture site, aligning with posterior border of mandibular ramus. While the long plate was stabilized from the submandibular incision site, screws through the trocar were fixed at the first and second hole of titanium plate onto superior condylar segment. Occlusion was checked and secured by wired intermaxillary fixation. Skipping the third and the fourth hole of titanium plate, the surgeons screwed the rest of the holes on the plate through submandibular incision site (Figure 2). After the long titanium plate was fixed, wound irrigation was done. Two 5 mm silicon Penrose drains were used for discharge drainage. Before the end of operation, the intermaxillary fixation wires were changed to rubbed bands. Liquid diet was recommended after 4 weeks postoperatively. The wires and arch bars were removed 4 weeks after the operation.

**FIGURE 3:**
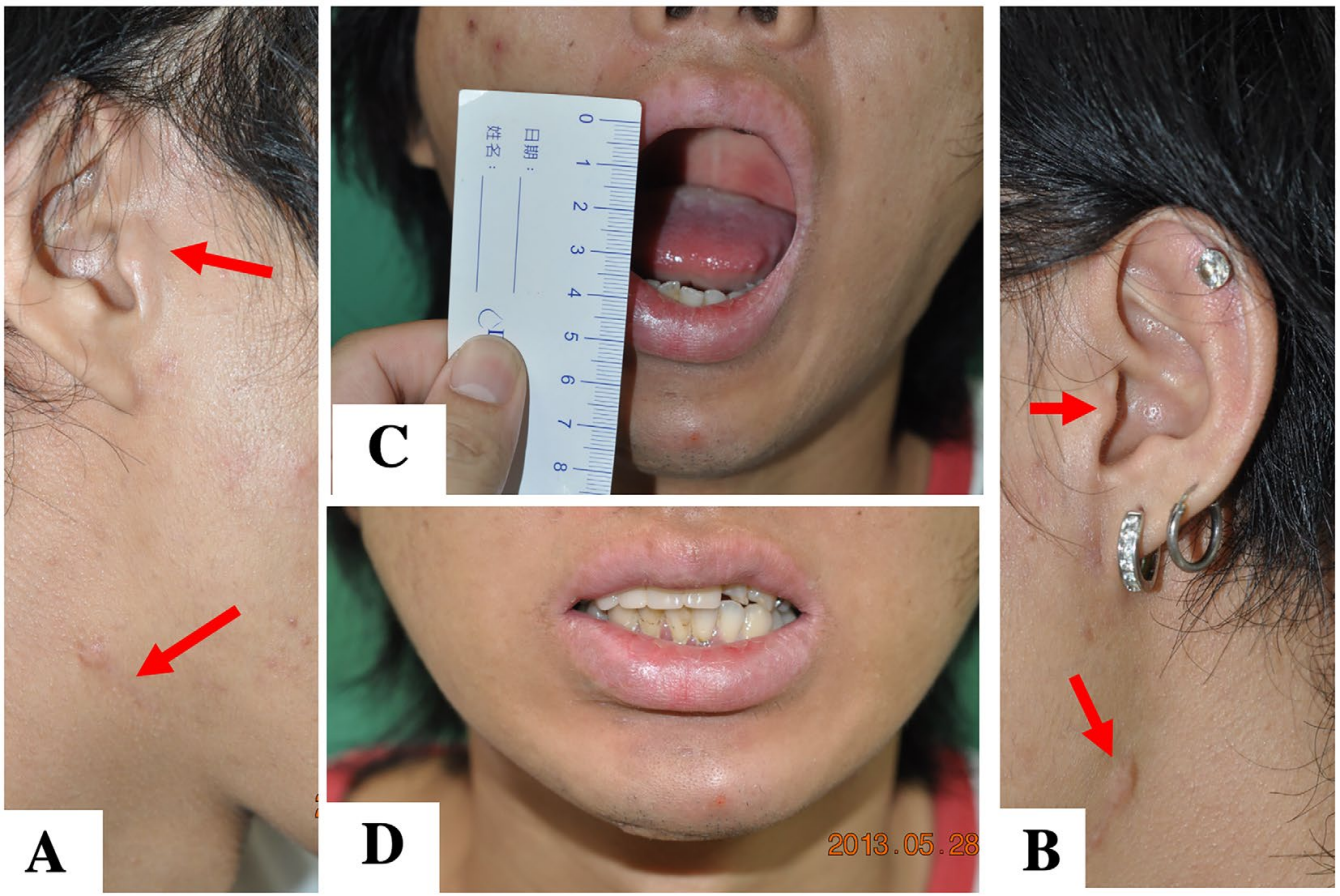
Postoperative pictures at the outpatient clinic 1 year and 4 months after operation. One of the panfacial fracture cases involved bilateral condylar fractures. The facial bone reconstruction operation lasted 815 min. The postoperative submandibular and preauricular scars (red arrow) were barely visible. (A) Right side; (B) left side, (C) mouth opening 35 mm shows no postoperative trismus. (D) No malocclusion, facial palsy or malalignment observed during operation.

**FIGURE 4:**
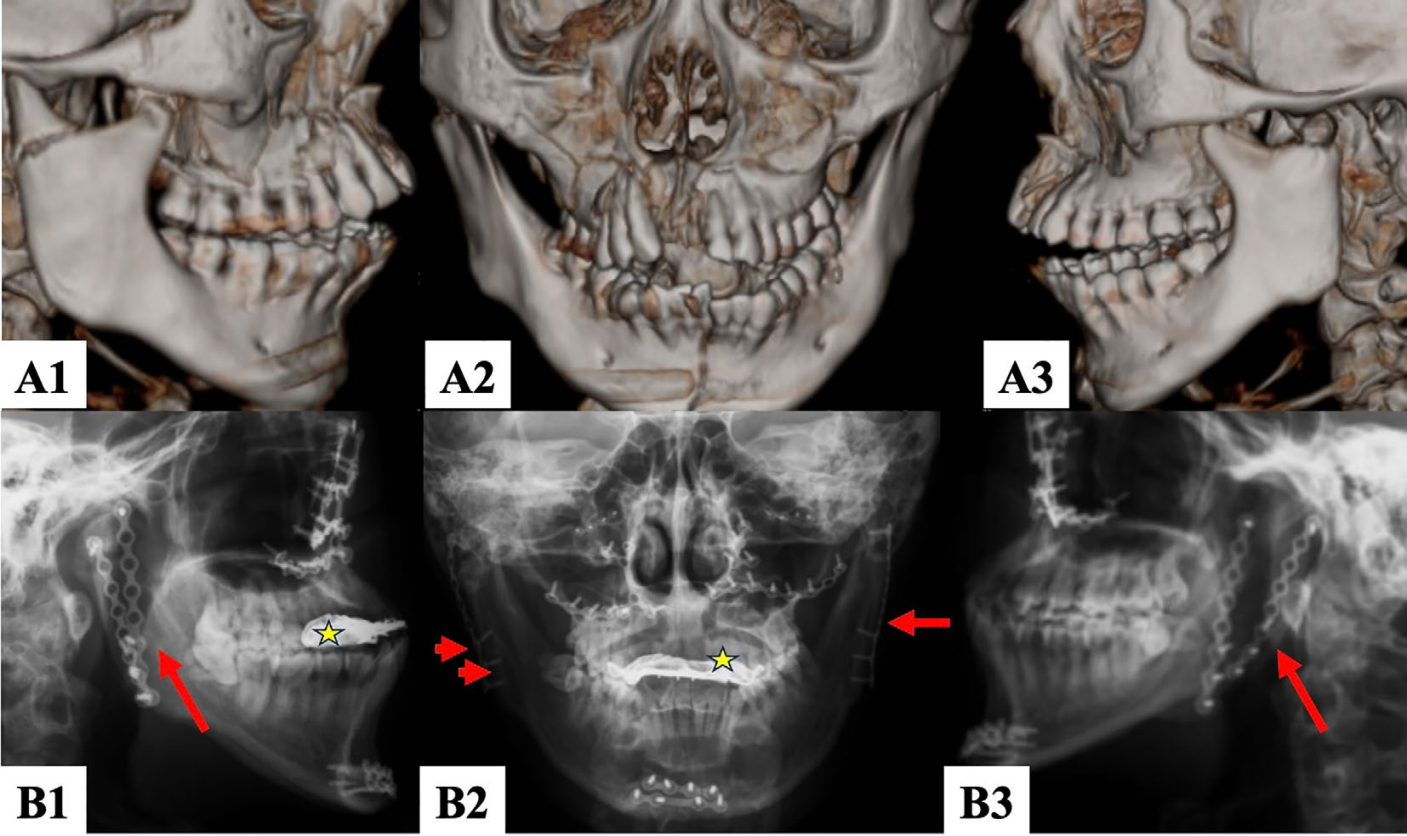
Application of Long Plate Technique to endoscopic approach of internal fixation. The same case presented in Figure 3. (A1–A3) Preoperative three dimensional (3D) reconstructive simulation suggested right orbital floor fracture, nasal bone fracture and bilateral subcondylar fracture, and mandibular fracture. (B1–B3) Postoperative radiographic pictures suggested bilateral condyle neck, maxilla and mandible open reduction and internal fixation with bilateral orbital floor reconstruction. Red arrow, an eight‐hole plate; Asterix: Upper dentures. During the radiographic exam, the upper dentures remained in place and were not part of the operation. They enhanced the patient's esthetics, while the surgery improved occlusion.

Postoperative follow‐ups were arranged on the first, second, and fourth weeks to record postoperative clinical conditions such as infection, hematoma, nerve injury, wound healing, facial scarring, facial symmetry, temporomandibular joint movement, occlusion, and mouth opening: 3.5–4 cm mouth opening and chin deviation (Figures 3 and 4).

## 3 Results

A total of 98 patients were included in this study (Table 1), with an average age of 32 years. The study cohort consisted of 52 males and 46 females. Within the studied population, 31 cases were classified under Group A, 25 cases were categorized within Group B, and 42 cases fell under Group C. Out of the total cases examined, 24 cases exhibited unilateral condylar fractures without associated facial bone fractures. Among these cases, 61 cases displayed unilateral condylar fractures alongside associated facial bone fractures. One case presented with bilateral condylar fractures without associated facial bone fractures, while 13 cases manifested bilateral condylar fractures along with associated facial bone fractures. The overall average operation time was 365 min. Specifically, the average operation time for Group A was 250 min, for Group B was 429 min, and for Group C was 413 min.

**TABLE 1:** Demographic and clinical characteristics of the patients (*n* = 98). IMF, intermaxillary fixation; short plate, three‐hole and four‐hole plate; long plate, six‐hole, seven‐hole, and eight‐hole plate.

	Group A (IMF + closed reduction)	Group B (IMF + short plate)	Group C (IMF + long plate)	Group A + B + C
Case number	31	25	42	98
Sex (female: male)	13:18	13:12	23: 20	48:50
Average age	36	31	30	32
**Diagnosis**				
Unilateral condylar fracture without associated facial bone fractures	11	6	7	24
Unilateral condylar fracture with associated facial bone fractures	14	18	29	61
Bilateral condylar fracture without associated facial bone fractures	1	0	0	1
Bilateral condylar fracture with associated facial bone fractures	5	1	7	13
Average intraoperative time (minutes) (minimal and maximum, mean ± standard deviation)	Mean: 250 (±139) Maximum: 420 Minimum: 45	Mean: 429 (±177) Maximum: 965 Minimum: 215	Mean: 413 (±137) Maximum: 815 Minimum: 155	Mean: 365 (±167) Maximum: 965 Minimum: 45

During regular follow‐ups (Table 2), four cases required reoperation due to mandibular dislocation (4/98, 4%): two cases from group A (2/30, 6.6%), one from group B (1/25, 4%), and one from group C (1/43, 2.3%). Malocclusion (4/98, 4%) was observed in three cases from group A (3/30, 10%) and one case from group C (1/43, 2.3%). In addition, four patients (5/98, 5.1%) experienced transient frontal branch weakness, one case in group B (1/25, 4%), and three cases in group C (4/3, 9.3%). The temporary weakness showed improvement by the 4th to 6th‐week follow‐up. No other complications were noted during the follow‐up period.

**TABLE 2:** Postoperation complications. Group A, IMF + closed reduction; Group B, IMF + short plate; Group C, IMF + long plate; IMF, intermaxillary fixation.

	Group A (IMF + closed reduction)	Group B (IMF + short plate)	Group C (IMF + long plate)	Group A + B + C
Case number	30	25	43	98
Reoperation	3	0	1	4
Mandible malocclusion	3	0	1	4
Frontal branch of the facial nerve transient weakness	0	1	4	5

## 4 Discussion

Management of mandibular subcondylar fracture remains controversial. The major challenge of the fracture site fixation is malalignment of the temporomandibular joint and facial nerve injury, which can affect articulation, food consumption, social function, and esthetic appearance. Miscellaneous approaches were developed to overcome this challenge [1, 14, 15, 16]. The retromandibular approach proposed by Girotti and Hinds [14] demonstrated excellent intraoperative visualization of the fracture site; however, visible scarring was observed. It is important to note that this approach is not suitable for addressing high locations of subcondylar fractures [15]. The endoscopic approach, on the other hand, was developed as a minimally invasive technique [16]. Muller et al. summarized experiences of the endoscopic approach and showed that the endoscopic approach, compared to open reduction, minimized significant scarring and reduced complication rates [16].

However, endoscopic‐assisted fixation can cause facial nerve traction injury, auricular anesthesia injury, and parotid gland trauma due to limited working space and a narrowed visual field, so there is a learning curve required for the surgeons. In addition, rigid fixation is generally achieved by a plate of the size of three‐hole or four‐hole, which can be troublesome while surgeons perform the insertion of screws into a plate. Given the constrained working space and restricted visibility at the fracture site, accommodating a plate, scope, and drill simultaneously poses challenges. Ensuring the stability of a three‐hole or four‐hole plate during drilling and screw insertion compounds the difficulty. Once the plate or screws slip off, searching for them can largely increase the operation time, which in turn increases the risk of iatrogenic injury to nerves and complications of anesthesia. Thus, the Long Plate Technique was developed to solve this dilemma. Surgeons can manipulate and maneuver the plate outside the working space using elongated Mosquito forceps, thereby circumventing the need to accommodate a plate, scope, and drill within a confined working area.

**FIGURE 5:**
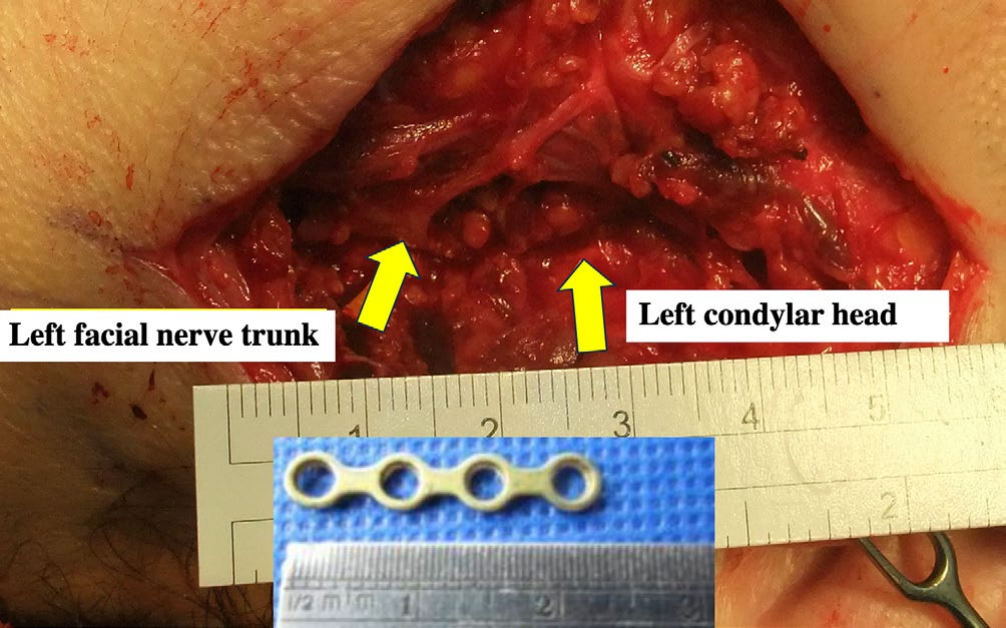
An intraoperative image illustrating the proximity of the third and the fourth holes of the plate to the facial nerve trunk.

In this study, the results showed that Group C had a lower average intraoperative time than Group B. Long Plate Technique can be conducted by insertion of a six‐hole, seven‐hole, or eight‐hole plate through submandibular incision. This method allows easy manipulation of the plate from submandibular incision. At the same time, the superior fractured segment of the condylar can be fixed with two screws through the trocar via the tragus incision. The third and fourth holes of the plate were very close to the trunk of the facial nerve (Figure 5). These two holes were skipped without screwing to prevent accidental facial nerve trunk injury (Figure 2). This technique is an innovation in endoscopic fixation of subcondylar fracture.

During regular follow‐ups (Table 2), reoperation due to temporomandibular joint dislocation occurred in four cases (4/98, 4%): two cases in Group A (2/30, 6.6%), one in Group B (1/25, 4%) and one case in Group C (1/43, 2.3%). These four patients were our early cases with less experienced skills. In addition, the long plate was fixed using only the first hole instead of both the first and second holes, leading to potential angulation and subsequent segmentation of the long plate in initial cases. The cases after these three presented with no complications during follow‐ups. Malocclusion was observed in four cases (4/98, 4%), three cases in Group A (3/30, 10%), and one case in Group C (1/43, 2.3%). In Group C, the patient experienced frequent seizure attacks and fell 3 days after the operation, resulting in a facial impact that led to malocclusion. The transient weakness of eyebrows was observed in four cases (5/98, 5.1%), one case in group B (1/25, 4%), and three cases in group C (4/43, 9.3%). This temporary weakness may stem from a traction injury to the frontal branch of the facial nerve. Such an injury can occur due to a close incision proximity to the facial nerve, inducing tension on the nerve when pulling the pretragus skin during the operation. These complications were not observed in the following fourth‐week to sixth‐week follow‐ups.

Several limitations were observed in this investigation. The study, being a single‐center retrospective analysis, is susceptible to potential selection biases. Due to the complexity and variety of facial bone fractures, categorizing them poses significant challenges for statistical analysis. Furthermore, the limited sample size necessitated the use of narrative statistics in this study. The inclusion of a restricted number of cases underscores the necessity for a larger sample size to mitigate the inherent risk of bias and enhance the study's robustness.

In conclusion, the sophisticated temporomandibular articulation allows slight malalignment, which remains the biggest challenge for surgeons [16]. Long Plate Technique provided a practical solution for anchoring the plate in cases of limited operational field during an endoscopic approach. This technique showed a reduction in operative time and may help decrease the risk of direct damage to the facial nerve, maxillary artery, and parotid gland. The Long Plate Technique offers a safe and efficient method that helps reduce intraoperative challenges for surgeons.

## Conflicts of Interest

The authors declare no conflicts of interest.

## Data Availability

The data that support the findings of this study are available from the corresponding author upon reasonable request.
